# The Functional and Prognostic Impact of TIGIT Expression on Bone Marrow NK Cells in Core Binding Factor-Acute Myeloid Leukemia Patients at Diagnosis

**DOI:** 10.3390/biomedicines12102207

**Published:** 2024-09-27

**Authors:** Dai-Hong Xie, Jun Wang, Kai Sun, Zong-Yan Shi, Ya-Zhe Wang, Yan Chang, Xiao-Ying Yuan, Yan-Rong Liu, Hao Jiang, Qian Jiang, Xiao-Jun Huang, Ya-Zhen Qin

**Affiliations:** Peking University People’s Hospital, Peking University Institute of Hematology, National Clinical Research Center for Hematologic Disease, Beijing Key Laboratory of Hematopoietic Stem Cell Transplantation, Beijing 100044, China

**Keywords:** TIGIT, NK cell function, core binding factor-acute myeloid leukemia, at diagnosis, prognosis, bone marrow

## Abstract

**Background**: The effect of the expression of the newly identified immune checkpoint, T cell immunoglobulin and immunoreceptor tyrosine-based inhibition motif domain (TIGIT) on NK cells in core binding factor-acute myeloid leukemia (CBF-AML) remains to be investigated. **Methods**: Fresh bone marrow samples from a total of 39 newly diagnosed CBF-AML patients and 25 healthy donors (HDs) were collected for testing the phenotype and function state of total NK, CD56^bright^, and CD56^dim^ NK cell subsets after in vitro stimulation. **Results**: The frequencies of TIGIT^+^ cells in total NK, CD56^bright^, and CD56^dim^ NK cell subsets had no significant difference between patients and HDs. TNF-α and INF-γ levels were uniformly lower in TIGIT^+^ cells than the corresponding TIGIT^−^ cells in all HDs, whereas those for TIGIT^+^ to TIGIT^−^ cells in patients were highly heterogenous; TIGIT expression was not related to PFP and GZMB expression in HDs, whereas it was related to higher intracellular PFP and GZMB levels in patients. Patients’ TIGIT^+^ NK cells displayed lower K562 cell-killing activity than their TIGIT^−^ NK cells. In addition, high frequencies of TIGIT^+^ cells in total NK and CD56^dim^ NK cells were associated with poor RFS. **Conclusions**: TIGIT expression affected the diagnostic bone marrow-sited NK cell function and had prognostic significance in CBF-AML patients.

## 1. Introduction

Acute myeloid leukemia (AML) is a heterogeneously hematologic malignancy that arises from the uncontrolled proliferation of myeloid blasts or progranulocytes [1,2]. Core binding factor-AML (CBF-AML) is defined as the presence of either t (8;21) (q22; q22) or inv (16) (p13q22)/t (16;16) (p13.1; q22) which individually result in the disruption of genes encoding subunits of CBF, a heterodimeric transcription factor essential in normal hematopoiesis [3,4]. CBF-AML accounts for about 15% of adult AML cases and is considered as a favorable-risk subtype [5]. Although adult CBF-AML patients own a higher complete remission (CR) rate, their relapse risk remains high and outcomes are heterogeneous [4,6,7,8]. Therefore, more novel prognostic biomarkers need to be identified for the improvement of risk stratification and risk-adapted therapies in CBF-AML patients.

Natural killer (NK) cells are a subset of lymphoid cells performing innate immunity functions [9,10,11]. In the tumor microenvironment (TME), the main function of NK cells is to kill tumor cells through perforin (PFP)/granzyme (GZMB) exocytosis and secrete an array of effector cytokines [9,12,13]. NK cells can be categorized into different subpopulations according to surface molecules and individual functions [9]. Based on surface antigen CD56, NK cells can be divided into the CD56^bright^ and CD56^dim^ subgroups [14,15]. CD56^bright^ NK cells mainly secrete cytokines such as tumor necrosis factor-α (TNF-α) and interferon-γ (IFN-γ) and CD56^dim^ NK cells mainly show cell-killing function [14,16,17]. NK cell is considered to hold promise in treating AML due to its strong function. On the other hand, AML malignant cells can develop several mechanisms to escape from NK cells’ immunity and make the therapeutic outcome even more challenging. The disrupted balance of costimulatory and coinhibitory receptors on NK cells’ surface is one of the important mechanisms for tumor cells’ immune escape [18]. The costimulatory and coinhibitory receptors shared a pair of ligands expressed on the surface of antigen-presenting cells (APCs) and tumor cells. The coinhibitory receptors can compete with its paired costimulatory receptors for ligand binding and thereby act as an antagonist of stimulation [19,20]. These paired receptors reciprocally regulate the immune responses of NK cells. The immune checkpoints are major components of coinhibitory receptors. The abnormal expression of immune checkpoints in NK cells may result in NK cell exhaustion displaying poor effector functions [13,21]. T cell immunoglobulin and immunoreceptor tyrosine-based inhibition motif domain (TIGIT) is one of the newly identified immune checkpoint molecules [22]. It could down-regulate NK cell function through interacting with its ligands, Poliovirus receptor (PVR, CD155), and family member nectin-2 (CD112), which are expressed on tumor cells. The TIGIT and its ligands were up-regulated in solid tumors and have shown their impact on patients’ outcomes [23,24,25,26,27]. With regard to AML, the expression pattern of TIGIT on NK cells in the peripheral blood and its prognostic significance has been reported but the results are controversial [28,29,30]. As for the bone marrow which was the TME of AML, just one study reported the expression pattern of TIGIT on NK cells, whereas its impact on patients’ outcomes has never been evaluated in either AML as a whole or AML subtypes [30].

In this study, we compared the expression level of TIGIT on total NK cells, CD56^bright^, and CD56^dim^ NK cell subsets in bone marrow between CBF-AML patients at diagnosis and healthy donors (HDs) by performing multi-parameter flow cytometry (MFC) testing. Thereafter, we analyzed the impact of its expression on NK cell function, patients’ treatment response, and clinical outcomes.

## 2. Materials and Methods

### 2.1. Patients and Treatment

This study recruited a total of 39 adult CBF-AML patients diagnosed at our institute from March to December 2021. The diagnosis was based on bone marrow morphology, immunophenotyping, karyotyping, and molecular biology [31]. Bone marrow samples collected from 29 patients at diagnosis were tested for NK cell phenotype and exerted survival analysis, and bone marrow mononuclear cells (BMMNCs) from 19 patients at diagnosis were obtained for NK cell function examination. This study also enrolled 25 HDs as controls. Bone marrow samples from 24 HDs were tested for NK cell phenotype and six HDs were exerted for NK cell function examination. The patients’ and HDs’ baseline characteristics are shown in Table 1.

The induction chemotherapy regimens included 1–2 cycles of AA (aclarubicin and cytarabine), HAA (homoharringtonine, aclarubicin and cytarabine), CAG (cytarabine, aclarubicin and granulocyte colony-stimulating factor), and VEN-AZA (venetoclax plus azacitidine). After achieving complete remission (CR), patients received either cytarabine-based chemotherapy alone or chemotherapy followed by allogeneic hematopoietic stem cell transplantation (allo-HSCT). Indications for allo-HSCT were described in our previous studies [32]. The cutoff date for the follow-up was January 2024.

The study was approved by the Ethics Committee of the Peking University People’s Hospital. Informed consent was obtained from all subjects in accordance with the Declaration of Helsinki.

### 2.2. NK Cell Phenotype Analysis

MFC was performed on fresh bone marrow aspirates. Briefly, samples were incubated with directly conjugated monoclonal antibodies CD45-V500 (BD Biosciences, Franklin Lakes, NJ, USA, Clone HI30), CD3-APC-H7 (BD Biosciences, Franklin Lakes, NJ, USA, Clone SK7), CD56-BV421 (Biolegend, San Diego, CA, USA, Clone HCD56), and TIGIT-PerCP-Cy5.5 (Biolegend, San Diego, CA, USA, Clone A15153G) for 15 min at room temperature. Then red blood cells (RBCs) were lysed using FACSTM lysing solution (BD Biosciences, Franklin Lakes, NJ, USA). After lysis, the cells were washed and resuspended in phosphate-buffered saline (PBS) (Solarbio, Beijing, China) followed by data acquirement on the FACSCanto™ (BD Biosciences, Franklin Lakes, NJ, USA). Approximately 1,000,000 events were collected at each test. Data analysis was performed with Kaluza 2.0 (Beckman Coulter, Brea, CA, USA).

The gating strategy is shown in Figure S1a–d. The FSC-H/FSC-A plot was used to exclude doublets and the FSC-A/SSC-A plot was performed to remove cellular debris and nonviable cells. Cells shown in the CD45/SSC-A plot were defined as nucleated cells (NCs). Lymphocytes were characterized as CD45-high/SSC-A-low in NCs. NK cells were the CD3-negative/CD56-positive cells. According to CD56 fluorescence intensity, NK cells were further divided into CD56^bright^ and CD56^dim^ NK cells. TIGIT^+^ and TIGIT^−^ cells were assessed in total NK cells, CD56^bright^, and CD56^dim^ NK cell subsets, respectively.

### 2.3. In Vitro NK Cell Stimulation and Function Testing

The BMMNCs were used to test the cytokine and cytotoxic-related molecule expression on NK cells. The BMMNCs were counted to 1 × 10^6^/mL which were stimulated with 2 × 10^5^/mL K562 target cells with an effector: target ratio of 5:1 supplemented with 2 μL/mL protein transport inhibitors (Invitrogen, Waltham, MA, USA) in the RPMI Medium 1640 (Gibco, Invitrogen, Paisley, Scotland, UK) containing 10% fetal bovine serum (FBS) (Gibco, Invitrogen, Paisley, Scotland, UK) and 1% penicillin/streptomycin (Gibco, Invitrogen, Paisley, Scotland, UK) in the 96 round bottom plate for 6 h at 37 °C. These cells were then stained with fluorochrome-labeled monoclonal antibodies CD45, CD3, CD56, and TIGIT. Thereafter, cells were permeabilized using the eBioscience Foxp3/Transcription Factor Staining Buffer Set (Invitrogen, Waltham, MA, USA) and intracellularly stained with anti-human TNF-α, IFN-γ, PFP, GZMB, and Ki67. The fluorescence of cells was tested on FACSCanto™ II and analyzed according to the protocol described above. As shown in Figure S1e–g, the expression levels of TNF-α, IFN-γ, PFP, GZMB, and Ki67 were assessed in total NK cells and its TIGIT^+^ and TIGIT^−^ subsets. The expression levels of TNF-α, IFN-γ, and Ki67 were assessed in CD56^bright^ NK cells and its TIGIT^+^ and TIGIT^−^ subsets. The expression levels of PFP, GZMB, and Ki67 were assessed in CD56^dim^ NK cells and its TIGIT^+^ and TIGIT^−^ subsets.

Frozen BMMNCs from CBF-AML patients were used for sorting TIGIT^+^ NK and TIGIT^−^ NK cells and evaluated NK-cell killing activity against K562 cells. BMMNCs were labeled with directly conjugated monoclonal antibodies including CD45-FITC, CD3-BV421, CD56-PE, and TIGIT-PerCP-Cy5.5 for 15 min at room temperature. BD FASC Aria III (BD Biosciences, Franklin Lakes, NJ, USA) was used for cell sorting. Thereafter, the TIGIT^+^ NK and TIGIT^−^ NK cells were individually co-cultured for 6 h at 37 °C with K562 cells with a ratio of 5:1, and 1000 IU/mL IL-2 was supplemented to maintain the viability of NK cells. Then apoptotic K562 cells were tested using the Apoptosis Detection Kit (LiankeBio, Hangzhou, Zhejiang, China).

The monoclonal antibodies used were TNF-α-PerCP-Cy5.5 (Biolegend, San Diego, CA, USA, Clone MAb11) and INF-γ-BV421 (Biolegend, San Diego, CA, USA, Clone B27), PFP-PerCP-Cy5.5 (Biolegend, San Diego, CA, USA, Clone B-D48), GZMB-BV421 (Biolegend, San Diego, CA, USA, Clone GB11), Ki67-BV421 (Biolegend, San Diego, CA, USA, Clone Ki-67), CD45-FITC (BD Biosciences, Franklin Lakes, NJ, USA, Clone HI30), CD3-BV421 (Biolegend, San Diego, CA, USA, Clone 17A2), CD56-PE (BD Biosciences, Franklin Lakes, NJ, USA, Clone B159) and TIGIT-PerCP-Cy5.5 (Biolegend, San Diego, CA, USA, Clone A15153G).

### 2.4. Definition and Statistical Analysis

CR referred to morphological CR with bone marrow blasts less than 5%, no extramedullary disease, and recovery of peripheral blood counts (neutrophils > 1 × 10^9^/L and platelets > 100 × 10^9^/L). Relapse-free survival (RFS) was measured from the date when CR was achieved to the date of relapse or the last bone marrow morphological evaluation. Overall survival (OS) was the period from the date of diagnosis to death from any cause or the final date they were last known to be alive.

The comparison of independent and paired variables was performed with the Mann–Whitney U test and Wilcoxon test. Fisher’s exact test was conducted to analyze categorical variables. Receiver operating characteristic (ROC) curves were used to find the optimal cutoff values based on the maximum Youden index. The Kaplan-Meier method and log-rank test were used to generate survival curves and to analyze the survival differences between different groups. A 2-sided *p* < 0.05 was considered statistically significant. Variables without multicollinearity and correlated with *p* < 0.05 in univariate analysis were selected for Cox multivariate analysis. All of the statistical analyses were performed using the SPSS 26.0 software package (SPSS Inc., Chicago, IL, USA) and GraphPad Prism 9 (GraphPad Software Inc., La Jolla, CA, USA).

## 3. Results

### 3.1. The Frequencies of TIGIT^+^ NK Cells in the Diagnostic Bone Marrows of CBF-AML Patients and Healthy Donors

**Figure 1:**
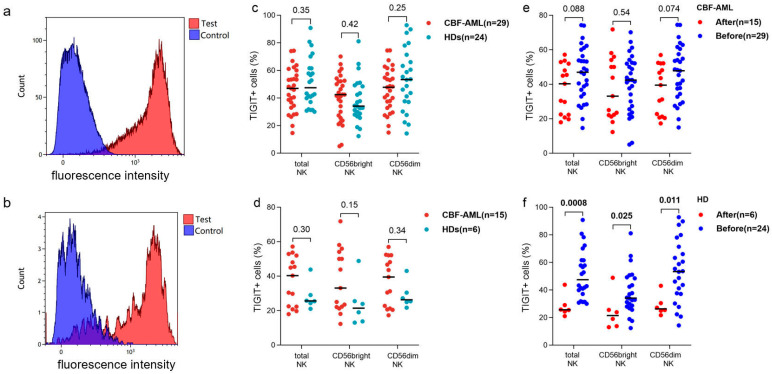
The expression pattern of TIGIT on NK cells of CBF-AML patients at diagnosis and HDs. (**a**) the representative histogram of TIGIT expression on CD56^dim^ NK cells of HD. (**b**) the representative histogram of TIGIT expression on CD56^bright^ NK cells of HD. (**c**) the expression pattern of TIGIT on NK cells between CBF-AML patients at diagnosis and HDs. (**d**) the expression pattern of TIGIT on NK cells between CBF-AML patients at diagnosis and HDs after stimulation in vitro. (**e**) the frequencies of TIGIT^+^ NK cells of CBF-AML patients before and after stimulation in vitro. (**f**) the frequencies of TIGIT^+^ NK cells of HDs before and after stimulation in vitro. CBF-AML: core binding factor-acute myeloid leukemia, HDs: healthy donors, MFI: mean fluorescence intensity. Numbers in this figure refer to the *p* values.

As shown in Figure 1c, the frequencies of TIGIT^+^ cells in total NK, CD56^bright^, and CD56^dim^ NK cell subsets had no significant difference between patients and HDs (median (range): 46.9% (14.7–74.3%) vs. 47.4% (30.1–90.8%), *p* = 0.35; 42.4% (5.0–70.2%) vs. 34.2% (12.4–81.2%), *p* = 0.42; 47.8% (15.0–74.5%) vs. 53.4% (14.3–92.8%), *p* = 0.25). In addition, the percentage of TIGIT^+^ CD56^bright^ NK cells was significantly lower than that in CD56^dim^ NK cells in HDs but not in patients (*p* = 0.0035 and 0.12) (Figure S2).

### 3.2. The Impact of Stimulation In Vitro on TIGIT Expression on NK Cells

In CBF-AML patients, the frequencies of TIGIT^+^ cells in total NK and CD56^dim^ NK cells after stimulation (*n* = 15) showed a tendency to be significantly lower compared with those of samples without stimulation (*n* = 29) (40.3% (17.9–57.2%) vs. 46.9% (14.7–74.3%), *p* = 0.088; 39.5% (17.3–57.0%) vs. 47.8% (15.0–74.5%), *p* = 0.074), but that in CD56^bright^ NK cells was similar (*p* = 0.54) (Figure 1e). Whereas in HDs, all the above three comparisons were significant (25.6% (21.0–43.8%) vs. 47.4% (30.1–90.8%), *p* = 0.0008; 21.5% (13.0–48.9%) vs. 34.2% (12.4–81.2%), *p* = 0.025; 26.3% (21.7–43.0%) vs. 53.4% (14.3–92.8%), *p* = 0.011) (Figure 1f). Likely, the pairwise comparison before and after stimulation of five patients and five HDs showed a similar trend (Figure S3). Even so, the frequencies of TIGIT^+^ cells in total NK, CD56^bright^, and CD56^dim^ NK subsets remained similar between patients and HDs after stimulation in vitro (*p* = 0.30, 0.15 and 0.34) (Figure 1d).

### 3.3. The Effect of TIGIT Expression on NK Cell Function in Patients and Healthy Donors

**Figure 2:**
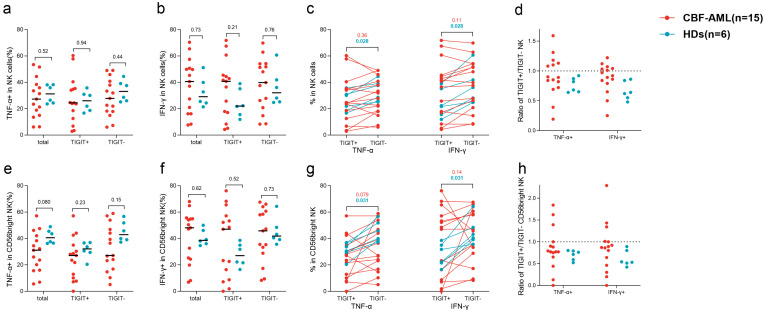
The impact of TIGIT expression on NK cell cytokine production in CBF-AML patients and HDs. TNF-α^+^ cells% in total NK cells (**a**), INF-γ+ cells% in total NK cells (**b**), TNF-α^+^ cells% and INF-γ^+^ cells% in pairing of TIGIT^+^ to TIGIT^−^ cells in total NK cells of individual cases (**c**), TNF-α^+^ cells% and INF-γ^+^ cells% ratios of TIGIT^+^/TIGIT^−^ NK cells (**d**), TNF-α^+^ cells% in CD56^bright^ NK cells (**e**), INF-γ^+^ cells% in CD56^bright^ NK cells (**f**), TNF-α^+^ cells% and INF-γ^+^ cells% in pairing of TIGIT^+^ to TIGIT^−^ cells in CD56^bright^ NK cells of individual cases (**g**), and TNF-α^+^ cells% and INF-γ^+^ cells% ratios of TIGIT^+^/TIGIT^−^ CD56^bright^ NK cells (**h**). Numbers in this figure refer to the *p* values.

We evaluated the cytokine production of NK cells and their CD56^bright^ subsets by examining their intracellular expression of TNF-α and IFN-γ after stimulation in vitro. We simultaneously evaluated their expression patterns shown by TIGIT^+^ to TIGIT^−^ paired comparison of each case and their expression levels in total NK, CD56^bright^ NK, and their TIGIT^+^, and TIGIT^−^ subsets between patients and HDs (Figure 2). The comparison showed that CD56^bright^ NK cells were the only group which had a tendency to have a lower percentage of TNF-α+ cells in patients than that in HDs (31.1% (5.6–57.1%) vs. 40.6% (36.2–49.0%), *p* = 0.080) (Figure 2a,b,e,f). For both total and CD56^bright^ NK cells in HDs, TIGIT^+^ cells were found to have significantly lower percentages of both TNF-α^+^ cells and INF-γ^+^ cells than the corresponding TIGIT^−^ cells (total: *p* = 0.028 and 0.028; CD56^bright^: *p* = 0.031 and 0.031) (Figure 2c,g). Whereas in patients, TIGIT^+^ cells tended to have a lower percentage of TNF-α+ cells than the corresponding TIGIT^−^ cells in CD56^bright^ subsets (*p* = 0.079), whereas no difference existed in the other three pair-comparisons (*p* = 0.36, 0.11, and 0.14) (Figure 2c,g). The TNF-α^+^ cells% and IFN-γ^+^ cells% ratios of TIGIT^+^/TIGIT^−^ total NK and CD56^bright^ NK cells of all six HDs were less than 1.0, whereas those patterns were highly varied in patients; 60.0% (9/15), 60.0% (9/15), 73.3% (11/15), and 66.7% (10/15) of patients individually had less than 1.0 of TIGIT^+^/TIGIT^−^ ratio for the TNF-α^+^ cells% in total NK, IFN-γ^+^ cells% in total NK, TNF-α^+^ cells% in CD56^bright^ NK, and IFN-γ^+^ cells% in CD56^bright^ NK cells, respectively (Figure 2d,h).

**Figure 3:**
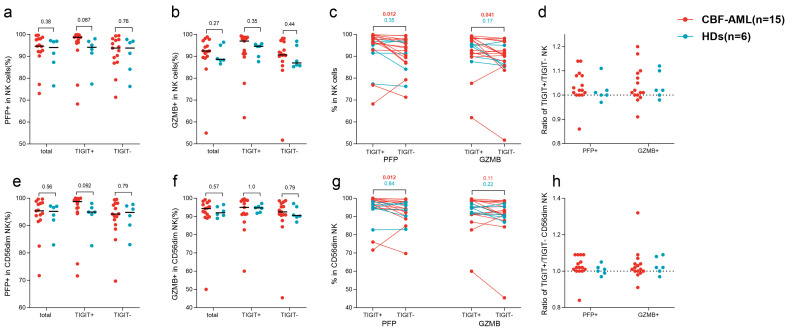
The impact of TIGIT expression on NK cell cytotoxicity in CBF-AML patients and HDs. PFP^+^ cells% in total NK cells (**a**), GZMB^+^ cells% in total NK cells (**b**), PFP^+^ cells% and GZMB^+^ cells% in pairing of TIGIT^+^ to TIGIT^−^ cells in total NK cells of individual cases (**c**), PFP^+^ cells% and GZMB^+^ cells% ratios of TIGIT^+^/TIGIT^−^ NK cells (**d**), PFP^+^ cells% in CD56^dim^ NK cells (**e**), GZMB^+^ cells% in CD56^dim^ NK cells (**f**), PFP^+^ cells% and GZMB+ cells% in pairing of TIGIT^+^ to TIGIT^−^ cells in CD56^dim^ NK cells of individual cases (**g**), and PFP^+^ cells% and GZMB^+^ cells% ratios of TIGIT^+^/TIGIT^−^ CD56^dim^ NK cells (**h**). Numbers in this figure refer to the *p* values.

NK cells’ cytotoxicity was assessed by the intracellular expression of PFP and GZMB after stimulation in vitro. For both total and CD56^dim^ NK cells in HDs, TIGIT^+^ cells had similar frequencies of both PFP^+^ and GZMB^+^ cells to the corresponding TIGIT^−^ counterparts (total NK: *p* = 0.35 and 0.17; CD56^dim^ NK: *p* = 0.84 and 0.22) (Figure 3c,f). Whereas in patients, TIGIT^+^ total NK cells had a significantly higher percentage of PFP+ and GZMB^+^ cells (*p* = 0.012 and 0.041) (Figure 3c). TIGIT^+^ CD56^dim^ NK cells had a significantly higher percentage of PFP+ cells compared with the corresponding TIGIT^−^ counterparts (*p* = 0.012) and that were similar in TIGIT^+^ CD56^dim^ NK cells (*p* = 0.11) (Figure 3f). Comparison of expression levels showed that the percentages of PFP+ cells in both TIGIT^+^ NK and TIGIT^+^ CD56^dim^ NK tended to be higher in patients than those in HDs (total: 98.7% (68.2–99.9%) vs. 94.1% (77.3–98.0%), *p* = 0.087; CD56^dim^: 98.8% (71.6–100.0%) vs. 94.9% (82.7–98.1%), *p* = 0.092) (Figure 3a,d) but other groups showed no significant difference (all *p* > 0.05) (Figure 3a,b,d,e). Differing from cytokine patterns, the distributions of PFP^+^ cells% and GZMB^+^ cells% ratios of TIGIT^+^/TIGIT^−^ were similar between patients and HDs (Figure 3d,h).

**Figure 4:**
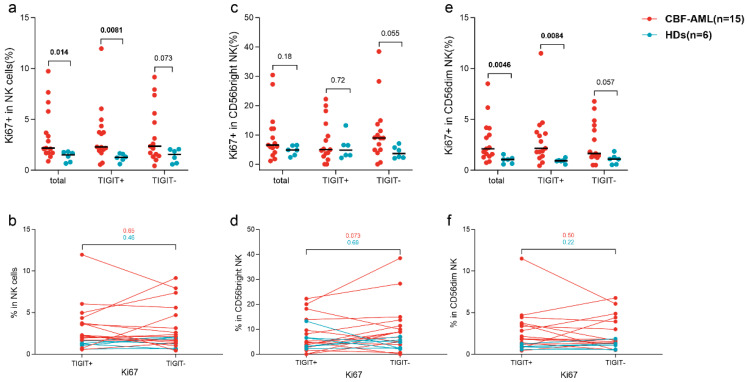
The impact of TIGIT expression on NK cell proliferation in CBF-AML patients and HDs. Ki67^+^ cells% in total NK cells (**a**), CD56^bright^ NK cells (**c**), and CD56^dim^ NK cells (**e**). Ki67+ cells% in pairing comparison of TIGIT^+^ to TIGIT^−^ cells in total NK cells (**b**), CD56^bright^ NK cells (**d**), and CD56^dim^ NK cells (**f**). Numbers in this figure refer to the *p* values.

We further evaluated the NK cells’ proliferation by testing Ki67 expression. Patients had or tended to have significantly higher rates of Ki67^+^ cells in total NK cells and CD56^dim^ NK cells and their TIGIT^+^ and TIGIT^−^ subsets compared with HDs (NK: 2.2% (0.88–9.7%) vs. 1.5% (0.67–1.8%), *p* = 0.014; 2.3% (0.56–12.0%) vs. 1.3% (0.61–1.7%), *p* = 0.0081; 2.4% (0.43–9.2%) vs. 1.5% (0.58–2.1%), *p* = 0.073; CD56^dim^ NK: 2.1% (0.74–8.5%) vs. 1.1% (0.58–1.6%), *p* = 0.0046; 2.2% (0.44–11.5%) vs 0.93% (0.57–1.3%), *p* = 0.0084; 1.7% (0.50–6.8%) vs. 1.1% (0.53–1.9%), *p* = 0.057) (Figure 4a,e). The percentages of Ki67^+^ cells in TIGIT^−^ CD56^bright^ NK cells tended to be higher in patients than that in HDs (*p* = 0.055) but no significant difference existed in CD56^bright^ NK and its TIGIT^+^ subsets (*p* = 0.18; *p* = 0.72) (Figure 4c). The paired comparison showed that just the percentage of Ki67^+^ cells in TIGIT^+^ CD56^bright^ NK cells in patients tended to be lower than corresponding TIGIT^−^ counterparts (*p* = 0.073) (Figure 4d).

**Figure 5:**
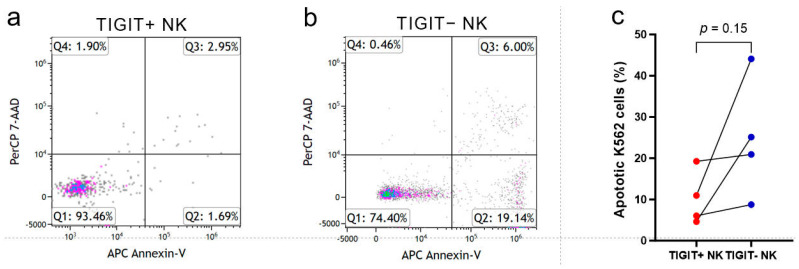
The impact of TIGIT expression on NK cell killing activity against K562 cells of four newly diagnosed CBFAML patients. The representative dot plots of the expression of apoptosis markers 7AAD and Annexin-V of K562 cells after co-culturing with TIGIT^+^ NK (**a**) and TIGIT^−^ NK cells (**b**). Pairwise comparison of apoptotic K562 cell proportion between TIGIT^+^ NK and TIGIT^−^ NK cells (**c**). The apoptotic K562 cell proportion is calculated by the addition of Q2 and Q3 (**a**,**b**). The dots in the subfigure (**a**,**b**) referred to the fluorescence intensity of each K562 cell. The red and blue dots in the subfigure (**c**) referred to the rates of apoptotic K562 cells co-cultured with TIGIT^+^ NK and TIGIT^−^ NK cells, respectively.

TIGIT^+^ NK and TIGIT^−^ NK cells sorted from four newly diagnosed CBF-AML patients were evaluated for K562 cell-killing activity (Figure 5a,b). Though the significant difference was not limited by the number of cases (*p* = 0.15), all four patients showed the same tendency: the proportion of apoptotic K562 cells in the TIGIT^+^ NK cells was lower than that in TIGIT^−^ NK cells (Figure 5c). The apoptotic K562 cells proportion ratios of TIGIT^−^ NK to TIGIT^+^ NK were 1.1, 1.4, 4.0, and 5.4, respectively. Therefore, TIGIT expression on NK cells was related to lower K562 cell-killing activity.

### 3.4. The Impact of TIGIT Expression on Bone Marrow NK Cells on Patient’s Outcomes

Of the 29 patients who were tested for NK cell phenotype at diagnosis, 23 (79.3%) received treatment and were followed up. Cr was achieved by 19 (82.6%) and 3 (13.0%) patients after 1 and 2 cycles of induction chemotherapy, respectively. Thereafter, 12 (52.2%) patients received chemotherapy alone and 10 (43.5%) patients received chemotherapy followed by allo-HSCT (matched sibling donor: *n* = 4; haploidentical related donor: *n* = 6) as consolidation. The median follow-up time for the entire cohort was 27.1 (range: 2.4–39.3) months. The 3-year RFS and OS rates were 79.7% (95% confidence interval [CI]: 54.5–91.9%) and 86.0% (95%CI: 62.5–95.3%), respectively.

As shown in Figure S4, the frequencies of TIGIT^+^ total NK, CD56^bright^, and CD56^dim^ NK cell subsets had no significant difference between patients who achieved CR and those who did not achieve CR after 1-course induction therapy (*p* = 0.29, 0.42, and 0.33). Furthermore, the percentage of TIGIT^+^ CD56^bright^ NK cells in relapsed patients tended to be significantly lower compared with that in patients in continuous CR (28.8% (5.0–43.9%) vs. 44.4% (6.0–70.2%), *p* = 0.098) and the frequencies of TIGIT^+^ cells in total NK and CD56^dim^ NK cells had no significant difference between patients relapsed and patients in continuous CR (*p* = 0.26 and 0.23) (Figure S5).

**Figure 6:**
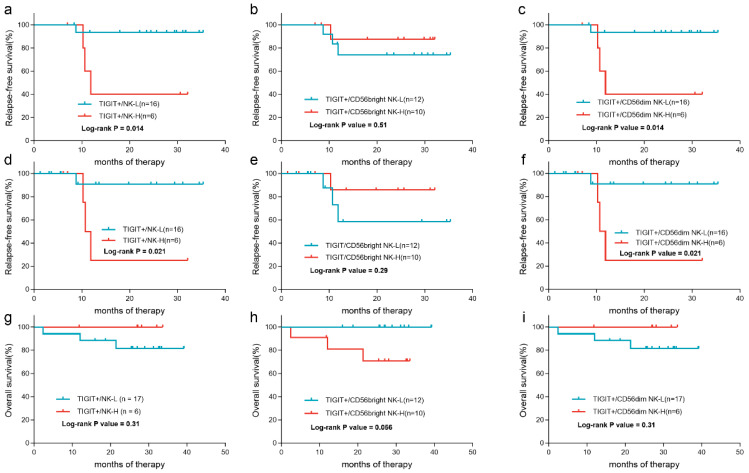
The impact of the expression of TIGIT on NK cells on patient outcomes. The RFS curves of patients grouped by the frequencies of TIGIT^+^ cells in total NK cells (**a**), CD56^bright^ NK cells (**b**), and CD56^dim^ NK cells (**c**). The RFS curves of patients censoring at the time of allo-HSCT grouped by the frequencies of TIGIT^+^ cells in total NK cells (**d**), CD56^bright^ NK cells (**e**), and CD56^dim^ NK cells (**f**). The OS curves of patients grouped by the frequencies of TIGIT^+^ cells in total NK cells (**g**), CD56^bright^ NK cells (**h**), and CD56^dim^ NK cells (**i**).

According to the ROC curves based on relapse, 55.1%, 43.0%, and 56.2% were the optimal cutoff values for the percentage of TIGIT^+^ cells in total NK cells, CD56^bright^, CD56^dim^ NK cell subsets to categorize patients into high (-H) and low (-L) groups (AUC = 0.69, *p* = 0.23; AUC = 0.78, *p* = 0.089; AUC = 0.71, *p* = 0.20), respectively (Figure S6). Patients with high percentages of TIGIT^+^ cells in total NK cells and CD56^dim^ NK cells had significantly poorer RFS than those with low percentages (3-year RFS rate: 40.0% (95%CI: 5.2–75.3%) vs. 93.3% (95%CI: 61.3–99.0%), *p* = 0.014; 40.0% (95%CI: 5.2–75.3%) vs. 93.3% (95%CI: 61.3–99.0%), *p* = 0.014) (Figure 6a,c), respectively, while the percentage of TIGIT^+^ cells in CD56^bright^ NK cells was not related to patient’ RFS rates (3-year RFS rate: 87.5% (95%CI: 38.7–98.1%) vs. 74.1% (95%CI: 39.1–90.9%), *p* = 0.51) (Figure 6b). Similarly, when censoring at the time of allo-HSCT, patients with high frequencies of TIGIT^+^ cells in total NK cells and CD56^dim^ NK cells had significantly lower RFS rates than those with low frequencies (3-year RFS rate: 25.0% (95%CI: 0.90–66.5%) vs. 90.9% (95%CI: 50.8–98.7%), *p* = 0.021; 25.0% (95%CI: 0.90–66.5%) vs. 90.9% (95%CI: 50.8–98.7%), *p* = 0.021) (Figure 6d,f). In contrast, the TIGIT^+^ cells in CD56^bright^ NK cells showed no correlation with RFS (3-year RFS rate: 85.7% (95%CI: 33.4–97.9%) vs. 58.3% (95%CI: 18.0–84.4%), *p* = 0.29) (Figure 6e). As for OS, patients with a high percentage of TIGIT^+^ cells in CD56^bright^ NK cells tended to have a significantly poorer OS compared with those with a low percentage (3-year OS rate: 70.7% (95%CI: 33.7–89.5%) vs. 100.0%, *p* = 0.056) (Figure 6h) while the other two groupings did not correlate with OS (*p* = 0.31 and 0.31) (Figure 6g,i).

### 3.5. Univariate and Multivariate Analysis of RFS and OS

Besides the frequencies of TIGIT^+^ cells in NK cells and its subsets, patients received chemotherapy alone as consolidation tended to be significantly associated with a shorter RFS compared with their corresponding control groups (3-year RFS rate: 63.6% (95%CI; 29.7–84.5%) vs. 100.0%, *p* = 0.054) (Table 2). In addition, patients who did not achieve CR after 1-course induction therapy had significantly poorer OS than those who achieved 1-course CR (3-year OS rate: 0 vs. 100.0%, *p* < 0.0001) (Table S1). We performed a multivariate Cox regression analysis for RFS that included the variables with *p* < 0.10 and without multicollinearity, and it showed that high frequency of TIGIT^+^ CD56^dim^ NK cells was the only independent predictor for poor RFS (HR: 10.1 (95%CI: 1.0–98.2), *p* = 0.045) (Table 2).

### 3.6. Relationship between TIGIT Expression on Bone Marrow NK Cells and Patients’ Clinical Characteristics and Molecular Abnormalities

As shown in Table S2, none of the frequencies of TIGIT^+^ cells in total NK cells and its subsets were correlated with gender, hemoglobin level, platelet counts, percentage of bone marrow blasts, and fusion genes (all *p* > 0.05). High white blood cell (WBC) counts tended to be related to a lower frequency of TIGIT^+^ cells in CD56^bright^ NK cells (*p* = 0.050) but were not associated with the frequencies of TIGIT^+^ cells in other cell groups (all *p* > 0.05). The frequencies of total NK and its three subsets had no significant difference between patients with c-KIT mutation and those without c-KIT mutation (all *p* > 0.05) (Figure S7). As for age, patients aged ≥40 years had significantly higher frequency of TIGIT^+^ cells in CD56^bright^ NK cells than those aged <40 years (48.9 (23.6–70.2) vs. 33.8 (5.0–61.2), *p* = 0.045), but no such significant difference existed in other groups (*p* = 0.37 and 0.37) (Figure S8).

## 4. Discussion

NK cells are one of the major cytotoxic effector cells of innate immunity that participate in the first line of defense against AML blast cells without antigen processing and presentation [33,34]. The over-expression of immune checkpoints is a hallmark of NK cell exhaustion. TIGIT, a novel immune checkpoint, can mediate inhibitory immunomodulatory regulation by interacting with its ligands CD155 and CD112 [35,36]. In the current study, we investigated the expression pattern of TIGIT in NK cells derived from bone marrow specimens of newly diagnosed CBF-AML patients and analyzed its impacts on NK cell function and patient outcomes. We found that the expression levels of TIGIT in total NK cells, CD56^bright^, and CD56^dim^ NK cell subsets were all similar between patients and HDs, whereas the impact of its expression on NK cell function was different. Survival analysis showed that high percentages of TIGIT^+^ cells in total NK and CD56^dim^ NK cells were associated with poorer RFS and a high percentage of TIGIT^+^ cells in CD56^bright^ NK cells tended to be related to poorer OS.

The up-regulated TIGIT expression on NK cells has been reported in solid tumors. Zhang et al. demonstrated that TIGIT expression was significantly higher in NK cells in intra-tumoral regions than those in peritumoral regions in patients with colon cancer [37]. Several studies revealed the expression levels of TIGIT in NK cells in the peripheral blood of newly diagnosed AML patients [28,29,30]. Valhondo et al. showed no significant difference in the percentage of NK cells expressing TIGIT between AML patients and HDs, while Liu et al. and Zeng et al. both observed that the frequencies of TIGIT^+^ cells in total NK cells, CD56^bright^, and CD56^dim^ NK cell subsets were all significantly higher in patients than those in healthy individuals [28,29,30]. The inconsistency may be attributed to the limited sample scales and different races of subjects enrolled. As for the bone marrow, we achieved the same findings as Zeng et al., that is, the proportion of TIGIT^+^ cells in total NK cells, CD56^bright^, and CD56^dim^ NK cell subsets in bone marrow was comparable between newly diagnosed AML patients and healthy individuals [30].

Thereafter we stimulated NK cells in vitro with K562 cells. The expression level of TIGIT in NK cells was downregulated after stimulation in HDs. Esen et al. also observed a similar phenomenon in healthy volunteers [38]. Sarhan et al. reported that NK cells stimulated with human cytomegalovirus (CMV) expressed lower levels of TIGIT compared to non-stimulated NK cells of CMV-seropositive healthy subjects [39]. This self-feedback phenomenon of NK cells in a physiological state indicated that TIGIT was indeed an inhibitory receptor negatively regulating NK cell function. In contrast to HDs, we found that the TIGIT expression on NK cells in patients was almost unresponsive to stimulation. The patients’ impaired self-feedback of TIGIT expression was consistent with their exhaustion status of NK cells when tumor antigens could not be actively dealt with.

We further comprehensively analyzed the effect of TIGIT expression on NK cell function. The direct in vitro K562 cell killing assay showed that TIGIT^+^ NK cells from all four patients uniformly had lower killing activity than their corresponding TIGIT^−^ NK cells, which demonstrated the inhibitory effect of TIGIT expression on NK cell-killing function in CBF-AML. As for cytokine production, the percentage of TNF-α-positive cells in CD56^bright^ NK cells tended to be lower in patients compared with HDs. What’s more, the TIGIT^+^ CD56^bright^ NK cells owned lower levels of TNF-α than TIGIT^−^ counterparts in patients. The patterns of TNF-α and IFN-γ ratios of TIGIT^+^/TIGIT^−^ suggested that the cytokine production of TIGIT^+^/TIGIT^−^ NK cells and CD56^bright^ NK cells were uniform in HDs but were highly heterogeneous in patients. With regard to NK cell’ cytotoxicity, TIGIT expression on total NK and CD56^dim^ NK cells was related to more PFP and GZMB intracellular accumulation in patients but not in HDs. TIGIT^+^ NK and CD56^dim^ NK cells not only had higher frequencies of PFP^+^ cells in patients than HDs but also had higher frequencies of PFP^+^ cells than TIGIT^−^ counterparts in patients. The percentage of GZMB+ cells in TIGIT^+^ NK cells was also higher than TIGIT^−^ NK cells in patients. The enhanced PFP/GZMB expression in the functionally exhausted lymphocytes has been observed in multiple studies on hematological malignancies. In the peripheral blood of newly diagnosed AML patients, Kong et al. found that PFP staining was elevated in TIGIT^+^ CD8^+^ T cells in peripheral blood [40]. Zhou et al. further demonstrated that CD26lowPD1^+^ CD8^+^ T cells with exhaustive features showed increased levels of PFP and GZMB than that in CD26^high^PD1^+^ CD8^+^ T cells in newly diagnosed AML [41]. Minnie et al. found that TIM-3^+^ in the bone marrow PD1^+^ CD8^+^ T cells of mice with relapsed myeloma was related to the enhanced gene expression of PFP and GZMB [42]. PFP and GZMB are stored in the cytoplasmic granules of NK cells [43,44]. Wherry et al. found that several genes involved in vesicle transport and regulation of the cytoskeleton were down-regulated in exhausted CD8^+^ T cells under chronic viral infections [45]. Therefore, the higher expression of PFP and GZMB in functionally exhausted lymphocytes just reflected the loss of their ability to secret PFP and GZMB granules effectively. We suspected that as a checkpoint molecule, TIGIT expression on NK cells affected cytotoxicity in AML by inhibiting the secreting of PFP and GZMB but had no such effect in HDs. The exact mechanisms behind the relationship between immune checkpoints and defective degranulation were still unknown.

It had been reported that TIGIT expression retained the capacity of proliferation of CD8^+^ T cells with higher levels of Ki67 expression and an increased proliferation index in the carboxyfluorescein-diacetate succinimidyl ester (CFSE) assay compared with TIGIT^−^ CD8^+^ T cells of newly diagnosed AML patients [40]. None of the studies had ever evaluated the effect of TIGIT on NK cell proliferation of AML patients. We found that the proliferation ability of TIGIT^+^ CD56^bright^ and CD56^dim^ NK cells in patients was not inferior to that in HDs. In both patients and HDs, TIGIT expression almost had no effect on the proliferation of NK cells.

Over-expressed TIGIT in tumor-infiltrating NK cells in solid tumors was reported to be prognostic [46]. As for AML, there were two studies which evaluated the prognostic significance of TIGIT on NK cells in the peripheral blood of newly diagnosed AML patients to date. Valhondo et al. demonstrated that the expression of TIGIT on NK cells had no significant effect on the OS of 36 newly diagnosed AML patients [29]. In accordance with this study, we found that the percentage of TIGIT^+^ NK cells in bone marrow was not associated with OS. The TIGIT expression on CD5^6bright^ NK cells but not CD56^dim^ NK cells was negatively related to patients’ OS. Considering the in vitro stimulation results, it suggested that impaired TNF-α production of TIGIT^+^ CD56^bright^ NK cells may be related to patient survival. The study by Liu et al. enrolled 23 newly diagnosed AML patients and showed that patients with a high frequency of TIGIT^+^ NK cells had a significantly high risk of poor prognosis risk grading [28]. The present study first found that a high percentage of TIGIT^+^ cells in bone marrow NK cells was associated with poorer RFS. Moreover, TIGIT on CD56^dim^ NK cells was an independent indicator of poor RFS accompanied by a loss of the ability to secrete PFP effectively. TIGIT on CD56^bright^ NK cells was not correlated with patients’ RFS, which may be related to the heterogeneous effect of TIGIT on cytokine production within them. It reminded us that further subgroup analysis was required.

## 5. Conclusions

The current study revealed that the frequencies of TIGIT^+^ cells in total NK cells, CD56^bright^, and CD56^dim^ NK cell subsets in the bone marrow of newly diagnosed CBF-AML patients were similar to that of HDs. The effect of TIGIT expression on NK cell function was different between patients and HDs. TNF-α and INF-γ levels were uniformly lower in TIGIT^+^ cells than their TIGIT^−^ cells in all HDs, whereas those for TIGIT^+^ to -cells in patients were highly heterogenous; TIGIT expression was not related to PFP and GZMB expression in HDs, whereas it was related to higher PFP and GZMB levels in patients. Furthermore, patients’ TIGIT^+^ NK cells displayed lower in vitro K562 cell killing activity than their TIGIT^−^ NK cells. In addition, high frequencies of TIGIT^+^ cells in total NK and CD56^dim^ NK cells were associated with poorer RFS and high frequencies of TIGIT^+^ cells in CD56^bright^ NK cells were associated with poorer OS. Although the number of cases enrolled is limited, this study does provide an important clue for the impacts of TIGIT expression on NK cell function state and a patient’s prognosis. Further studies in larger cohorts of patients are warranted to confirm the conclusions.

## Figures and Tables

**Table 1:** The baseline characteristics of enrolled patients and HDs.

Variables	NK Cell Function Group		NK Cell Phenotype Group	
	Patients	HDs	Patients	HDs
Number of cases	19 ^1^	6	29	24
Age (year)	38 (17–51)	53 (36–57)	39 (17–62)	47 (31–65)
Gender (male/female)	12/7	5/1	11/18	16/8
t(8;21)/RUNX1::RUNX1T1 (*n*)	19	NA	25	NA
inv(16)/CBFB::MYH11 (*n*)	0	NA	4	NA
c-KIT mutation (*n*)	10	NA	14	NA
WBC count (×10^9^/L)	14.5 (4.0–50.1)	6.5 (3.7–6.9)	9.9 (0.85–90.8)	6.1 (3.7–7.9)
Hemoglobin (g/L)	73.0 (36.0–130.0)	155.5 (127.0–161.0)	75.0 (43.0–144.0)	149.5 (121.0–185.0)
Platelet count (×10^9^/L)	28.0 (8.0–106.0)	204.0 (137.0–303.0)	27.0 (3.0–134.0)	223.5 (137.0–336.0)
BM blast percentage (%)	46.0 (23.0–90.0)	NA	55.0 (12.0–89.0)	NA

^1^ The continuous variables were represented by the median (range) and the categorical variables were represented by the number of cases.

**Table 2:** Univariate and multivariate analysis of variables on RFS in the entire cohort (*n* = 22).

Variables	Univariate Analysis		Multivariate Analysis	
	3-Year RFS Rate (95%CI)	*p* Value	HR (95%CI)	*p* Value
**TIGIT^+^/NK (%)**		**0.014**	—	—
≤55.1 (*n* = 16)	93.3% (61.3–99.0%)			
>55.1 (*n* = 6)	40.0% (5.2–75.3%)			
TIGIT^+^/CD56^bright^ NK (%)		0.51		
≤43.0 (*n* = 12)	74.1% (39.1–90.9%)			
>43.0 (*n* = 10)	87.5% (38.7–98.1%)			
**TIGIT^+^/CD56^dim^ NK (%)**		**0.014**		**0.045**
≤56.2 (*n* = 16)	93.3% (61.3–99.0%)		1.0	
>56.2 (*n* = 6)	40.0% (5.2–75.3%)		10.1 (1.0–98.2)	
Age (year)		0.51		
<40 (*n* = 14)	74.1% (39.1–90.9%)			
≥40 (*n* = 8)	87.5% (38.7–98.1%)			
Gender		0.76		
Male (*n* = 8)	75.0% (31.5–93.1%)			
Female (*n* = 14)	83.3% (48.2–95.6%)			
WBC counts (×10^9^/L)		0.28		
≤10.0 (*n* = 11)	90.0% (47.3–98.5%)			
>10.0 (*n* = 11)	68.6% (30.5–88.7%)			
Hemoglobin (g/L)		0.41		
≤76.0 (*n* = 10)	88.9% (43.3–98.4%)			
>76.0 (*n* = 12)	71.6% (35.0–89.9%)			
Platelet counts (×10^9^/L)		0.30		
≤27.0 (*n* = 11)	90.0% (47.3–98.5%)			
>27.0 (*n* = 11)	70.0% (32.9–89.2%)			
BM blast percentage (%)		0.18		
≤55.0 (*n* = 13)	90.9% (50.8–98.7%)			
>55.0 (*n* = 9)	64.8% (25.3–87.2%)			
KIT mutation		0.25		
Negative (*n* = 12)	70.0% (32.9–89.2%)			
Positive (*n* = 10)	88.9% (43.3–98.4%)			
Consolidation regimen		0.054	—	0.15
Chemotherapy only (*n* = 12)	63.6% (29.7–84.5%)			
Allo-HSCT (*n* = 10)	100.0%			
CR after 1-course induction		0.15		
No (*n* = 3)	50.0% (0.60–91.0%)			
Yes (*n* = 19)	83.0% (55.9–94.2%)			

Bold text refers to the variables with *p* < 0.05.

## Data Availability

The data underlying this article are available from the corresponding author upon their reasonable request.

## Supplementary Materials

The following supporting information can be downloaded at: https://www.mdpi.com/article/10.3390/biomedicines12102207/s1, Figure S1: The gating strategy of MFC for lymphocytes (a), NK cells (b), CD56^bright^ and CD56^dim^ NK cells (c), TIGIT^+^ and TIGIT^−^ cells in total NK cells, CD56^bright^, and CD56^dim^ NK cell subsets (d), and TNF-α^+^, IFN-γ^+^, PFP^+^, GZMB^+^ and Ki67^+^ cells in total NK cells and its TIGIT^+^ and TIGIT^−^ subsets (e), TNF-α^+^, IFN-γ^+^, and Ki67^+^ cells in CD56^bright^ NK cells and its TIGIT^+^ and TIGIT^−^ subsets (f), and PFP^+^, GZMB^+^, and Ki67^+^ cells in CD56^dim^ NK cells and its TIGIT^+^ and TIGIT^−^ subsets (g); Figure S2: The pairwise comparison of TIGIT expression on CD56^bright^ and CD56^dim^ NK cell subsets of CBF-AML patients and HDs. Numbers in this figure refer to the *p* values; Figure S3: The pairwise comparison of TIGIT expression on NK cells before and after stimulation in vitro of CBF-AML patients and HDs. Numbers in this figure refer to the *p* values; Figure S4: Comparison of the frequencies of TIGIT^+^ NK cells between patients who achieved CR and those who did not achieve CR after 1-course induction therapy. Numbers in the figure refer to the *p* values; Figure S5: Comparison of the frequencies of TIGIT^+^ NK cells between patients who relapsed and those who remained continuous CR. Numbers in the figure refer to the *p* values; Figure S6: The ROC curve analysis of the percentage of TIGIT^+^ cells in total NK cells (a), CD56^bright^ NK cells (b), and CD56^dim^ NK cells (c); Figure S7: The expression levels of TIGIT on total NK cells, CD56^bright^ NK, and CD56^dim^ NK in patients with and without c-KIT mutation. Numbers in the figure refer to the *p* values; Figure S8: The expression levels of TIGIT on total NK cells, CD56^bright^, and CD56^dim^ NK cell subsets in patients aged ≥40 (*n* = 13) and <40 (*n* = 16). Numbers in the figure refer to the *p* values. Table S1: Univariate analysis of variables on OS in the entire cohort (*n* = 23); Table S2: The relationship between TIGIT expression in NK cells and patients’ clinical characteristics and molecular abnormalities.
