# Supplementary material for: The Functional and Prognostic Impact of TIGIT Expression on Bone Marrow NK Cells in Core Binding Factor-Acute Myeloid Leukemia Patients at Diagnosis
Source: Biomedicines. 2024 Sep 27;12(10):2207. doi: 10.3390/biomedicines12102207 (PMC11504867; doi:10.3390/biomedicines12102207)
Supplement: Supplementary file 1 [file biomedicines-12-02207-s001.zip › biomedicines-3099357-supplementary.pdf]

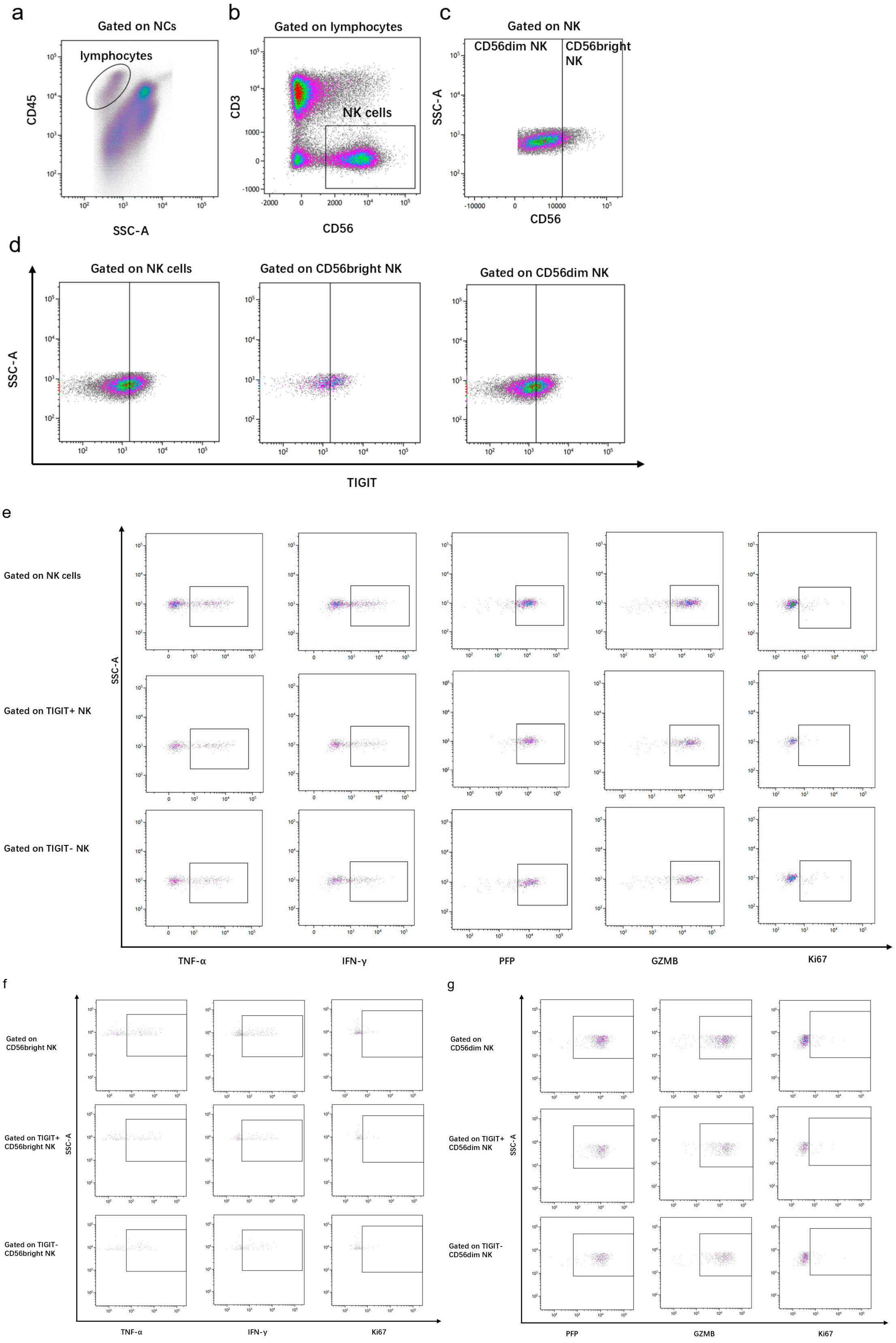

Figure S1. The gating strategy of MFC for lymphocytes (**a**), NK cells (**b**), $CD56^{bright}$ and $CD56^{dim}$ NK cells (**c**), $TIGIT^{+}$ and $TIGIT^{-}$ cells in total NK cells, $CD56^{bright}$, and $CD56^{dim}$ NK cell subsets (**d**), and TNF-α^{+}, IFN-γ^{+}, PFP^{+}, $GZMB^{+}$ and $Ki67^{+}$ cells in total NK cells and its TIGIT+ and TIGIT- subsets (**e**), TNF-α^{+}, IFN-γ^{+}, and $Ki67^{+}$ cells in $CD56^{bright}$ NK

cells and its TIGIT^{+} and TIGIT^{-} subsets (**f**), and PFP^{+}, GZMB^{+}, and Ki67^{+} cells in CD56dim NK cells and its TIGIT^{+} and TIGIT^{-} subsets (**g**).

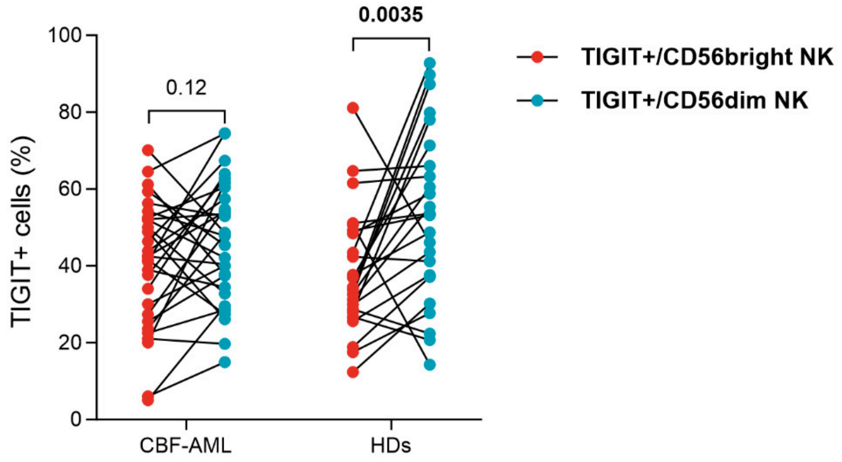

Figure S2. The pairwise comparison of TIGIT expression on CD56^{bright} and CD56^{dim} NK cell subsets of CBF-AML patients and HDs. Numbers in this figure refer to the *p* values.

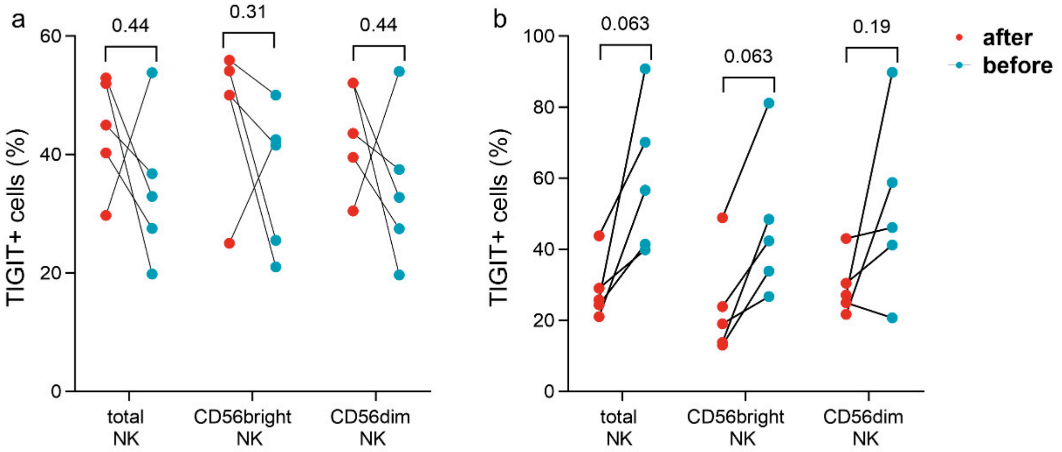

Figure S3. The pairwise comparison of TIGIT expression on NK cells before and after stimulation in vitro of CBF-AML patients and HDs. Numbers in this figure refer to the *p* values.

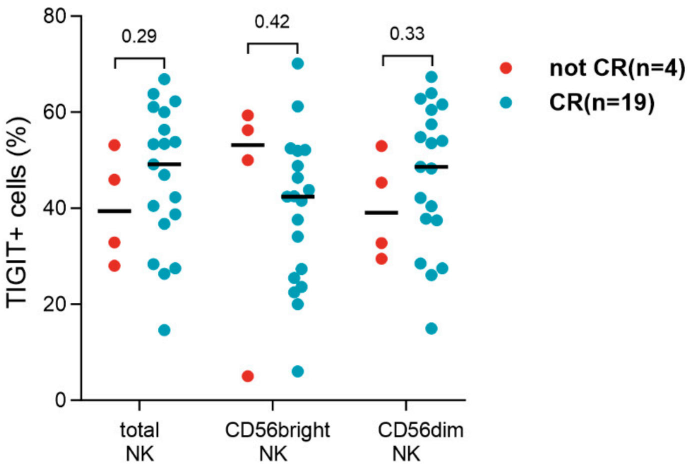

Figure S4. Comparison of the frequencies of TIGIT⁺ NK cells between patients who achieved CR and those who did not achieve CR after 1-course induction therapy. Numbers in the figure refer to the *p* values.

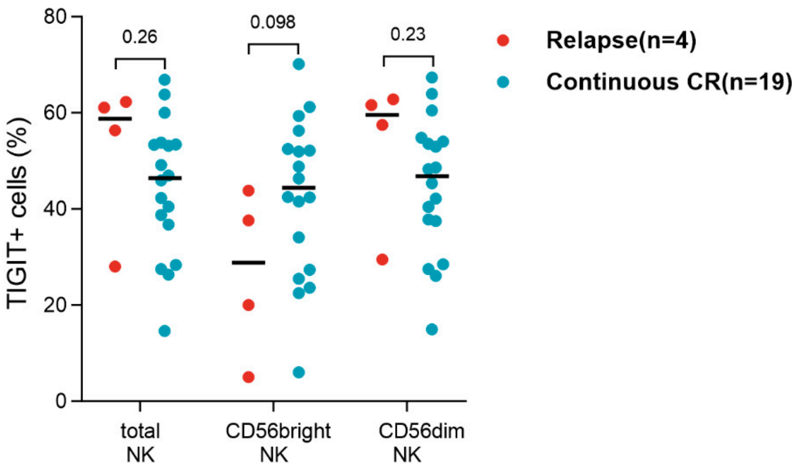

Figure S5. Comparison of the frequencies of TIGIT⁺ NK cells between patients who relapsed and those who remained continuous CR. Numbers in the figure refer to the *p* values.

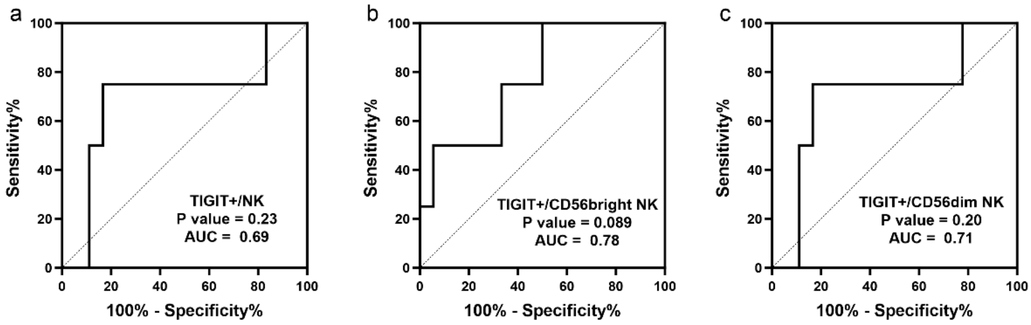

Figure S6. The ROC curve analysis of the percentage of TIGIT$^+$ cells in total NK cells (**a**), CD56bright NK cells (**b**), and CD56dim NK cells (**c**).

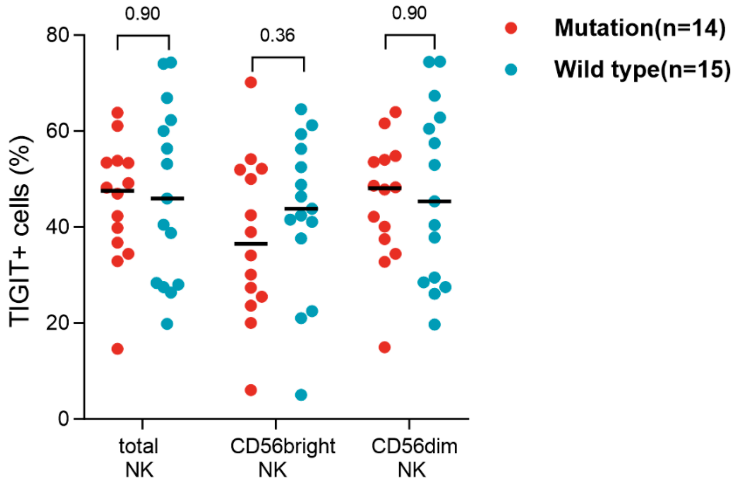

Figure S7. The expression levels of TIGIT on total NK cells, CD56^{bright} NK, and CD56dim NK in patients with and without c-KIT mutation. Numbers in the figure refer to the *p* values.

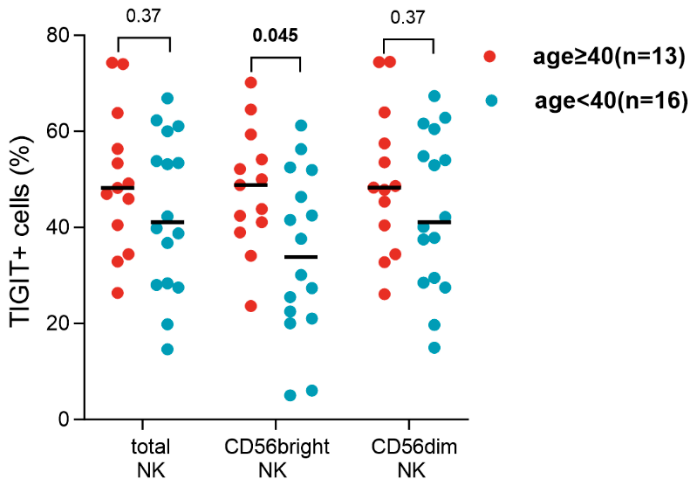

Figure S8. The expression levels of TIGIT on total NK cells, CD56^{bright}, and CD56^{dim} NK cell subsets in patients aged ≥ 40 (n = 13) and < 40 (n = 16). Numbers in the figure refer to the *p* values.

Table S1. Univariate analysis of variables on OS in the entire cohort (n = 23).

**Variables**	**Univariate Analysis**	
	**3-year OS Rate (95%CI)**	***p* Value**
TIGIT$^+$/NK (%)		0.31
≤55.1 (n = 17)	81.4% (52.6%-93.7%)	
>55.1 (n = 6)	100.0%	
TIGIT$^+$/CD56^{bright} NK (%)		0.056
≤43.0 (n = 12)	100.0%	
>43.0 (n = 11)	70.7% (33.7%-89.5%)	
TIGIT+/CD56^{dim} NK (%)		0.31
≤56.2 (n = 17)	81.4% (52.6%-93.7%)	
>56.2 (n = 6)	100.0%	
Age (year)		0.36
<40 (n = 14)	92.3% (56.6%-98.9%)	
≥40 (n = 9)	77.8% (36.5%-93.9%)	
Gender		0.80
Male (n = 9)	88.9% (43.3%-98.4%)	
Female (n = 14)	83.1% (47.2%-95.5%)	
WBC counts (×10^9/L)		0.52
≤10.0 (n = 11)	90.9% (50.8%-98.7%)	
>10.0 (n = 12)	80.2% (40.3%-94.8%)	
Hemoglobin (g/L)		0.45
≤76.0 (n = 11)	79.5% (39.3%-94.5%)	
>76.0 (n = 12)	91.7% (53.9%-98.8%)	
Platelet counts (×10^9/L)		0.56
≤27.0 (n = 12)	81.5% (43.5%-95.1%)	
>27.0 (n = 11)	90.9% (50.8%-98.7%)	
BM blast percentage (%)		0.38
≤55.0 (n = 13)	91.7% (53.9%-98.8%)	
>55.0 (n = 10)	77.1% (34.5%-93.9%)	
KIT mutation		0.60
Negative (n = 12)	80.8% (42.4%-94.9%)	
Positive (n = 11)	90.9% (50.8%-98.7%)	
Consolidation regimen (n = 22)		0.13
Chemotherapy only (n = 12)	100.0%	
Allo-HSCT (n = 10)	78.8% (38.1%-94.3%)	
**CR after 1-course induction**		**< 0.0001**
No (n = 4)	0	
Yes (n = 19)	100.0%	

Table S2. The relationship between TIGIT expression in NK cells and patients' clinical characteristics and molecular abnormalities.

**Variables**	**TIGIT$^+$/NK (%)**	***p* Value**	**TIGIT$^+$/CD56^{bright} NK (%)**	***p* Value**	**TIGIT$^+$/CD56^{dim} NK (%)**	***p* Value**	**TIGIT$^+$/CD56^{dim} CD57$^+$ NK (%)**	***p* Value**
Age (year)		0.37		**0.045**		0.37		0.95
<40 (n = 16)	41.1 (14.7-66.9)		33.8 (5.0-61.2)		41.1 (15.0-67.4)		49.7 (15.4-75.8)	
≥40 (n = 13)	48.2 (26.4-74.3)		48.9 (23.6-70.2)		48.3 (26.1-74.5)		45.7 (22.3-78.8)	
Gender		0.59		0.79		0.69		1.0
Male (n = 11)	42.3 (28.4-74.3)		42.4 (20.0-64.6)		42.1 (28.5-74.4)		40.0 (28.2-74.0)	
Female (n = 18)	48.0 (14.7-74.0)		42.0 (5.0-70.1)		48.5 (14.9-74.5)		48.8 (15.4-78.8)	
WBC count (×10^9/L)		0.22		0.050		0.21		0.21
≤10.0 (n = 15)	53.1 (19.8-74.3)		48.9 (21.0-70.1)		53.0 (19.7-74.5)		55.8 (22.3-78.8)	
>10.0 (n = 14)	41.4 (14.7-66.9)		38.3 (5.0-59.4)		41.4 (14.9-67.4)		45.3 (15.4-75.8)	
Hemoglobin (g/L)		0.76		0.34		0.79		0.69
≤75.0 (n = 15)	46.0 (14.7-74.3)		46.3 (6.0-70.1)		45.4 (14.9-74.5)		45.1 (15.4-78.8)	
>75.0 (n = 14)	50.7 (26.4-63.8)		40.0 (5.0-61.2)		50.4 (26.1-64.0)		52.0 (22.3-65.7)	
Platelet count (×10^9/L)		0.63		0.46		0.60		0.93
≤27.0 (n = 15)	49.1 (14.7-74.0)		43.9 (6.0-70.1)		48.6 (14.9-74.5)		46.0 (15.4-78.8)	
>27.0 (n = 14)	40.2 (26.4-74.3)		38.3 (5.0-64.6)		40.3 (26.1-74.4)		46.7 (22.3-74.0)	
Bone marrow blast percentage (%)		0.69		0.57		0.60		0.83
≤55.0 (n = 15)	49.1 (14.7-74.0)		42.4 (6.0-70.1)		48.6 (14.9-74.5)		45.7 (15.4-78.8)	
>55.0 (n = 14)	44.1 (19.8-74.3)		40.1 (5.0-64.6)		43.8 (19.7-74.4)		48.3 (28.2-74.0)	
Fusion genes		0.61		0.21		0.66		0.90
RUNX1-RUNX1T1 (n = 25)	46.9 (14.7-74.3)		42.5 (5.0-70.1)		47.8 (14.9-74.5)		46.0 (15.4-78.8)	
CBFB-MYH11 (n = 4)	51.7 (28.4-62.3)		30.1 (20.0-52.0)		51.9 (28.5-62.8)		48.3 (28.2-65.7)	
c-KIT mutation		0.90		0.36		0.90		0.57
Mutation (n = 14)	47.6 (14.7-63.8)		36.5 (6.0-70.1)		48.1 (14.9-64.0)		45.8 (15.4-72.6)	
Wild type (n = 15)	46.0 (19.8-74.3)		43.9 (5.0-64.6)		45.4 (19.7-74.5)		53.4 (22.3-78.8)	
